# Morphogenetic Variability and Hypertension in Ischemic Stroke Patients—Preliminary Study

**DOI:** 10.3390/jcm7070162

**Published:** 2018-06-26

**Authors:** Milan Savic, Suzana Cvjeticanin, Milica Lazovic, Ljubica Nikcevic, Dejan Nikolic

**Affiliations:** 1 Special Hospital for Cerebrovascular Diseases “Sveti Sava”, Belgrade 11000, Serbia; milan.savic@svetisava.rs (M.S.); ljubicanikcevic@yahoo.com (L.N.); 2 Institute for Human Genetics, Faculty of Medicine, University of Belgrade, Belgrade 11000, Serbia; cujasimsi@gmail.com; 3 Faculty of Medicine, University of Belgrade, Belgrade 11000, Serbia; lazovicmilica15@gmail.com; 4 Institute for Rehabilitation, Belgrade 11000, Serbia; 5 Department of Physical Medicine and Rehabilitation, University Children’s Hospital, Tirsova 10, Belgrade 11000, Serbia

**Keywords:** manifested ischemic stroke, homozygous recessive characteristics, variability, hypertension

## Abstract

In this study, we evaluated and compared the morphogenetic variability and the degree of recessive homozygosity in patients with manifested ischemic stroke compared to healthy controls. We have evaluated 120 patients with manifested ischemic stroke, of which 64 did not have hypertension and 56 have hypertension. For comparison, we additionally tested 194 healthy individuals without manifested ischemic stroke (controls). For the estimation of the degree of recessive homozygosity, we have performed the homozygously recessive characteristics (HRC) test and tested 19 HRCs. There was a significant difference in the individual variations of 19 HRCs between the controls and patients with manifested ischemic stroke (∑χ^2^ = 60.162, *p* < 0.01). The mean values of the tested HRCs significantly differed between the controls and group with manifested ischemic stroke (Controls − 5.71 ± 1.61, Ischemic stroke group − 6.25 ± 1.54, *p* = 0.012). For the tested individuals with hypertension, the mean values of HRCs did not significantly differ between the controls and those that had manifested ischemic stroke (Controls − 5.28 ± 1.75, Ischemic stroke group − 5.64 ± 1.48, *p* = 0.435). We found a significant difference in the frequencies of HRCs between those with and without hypertension for controls (*p* < 0.003) and for those with manifested ischemic stroke (*p* < 0.001). There are increased degrees of recessive homozygosity along with decreased variability in patients with manifested ischemic stroke compared to controls.

## 1. Introduction

In the developed world, stroke is considered to be the leading cause of long-term disability and the third most common cause of death [1,2]. The exact role of genetics is still controversial in stroke patients due to the heterogeneous etiopathogenesis [1]. However, numerous monogenic disorders have been described to be associated with the stroke, including those on autosomal, sex and mitochondrial chromosomes as well as polygenic disorders [1,2,3]. These single gene disorders, which have a lower prevalence rate in individuals that are carriers of mutations, are associated with a higher risk for stroke [4]. Thus far, several locations on different chromosomes have been associated with the occurrence of ischemic stroke (1q24.2, 7q36.1, 11p11.2, 13q12.3 and 14q23.1 OMIM 601367) and with hemorrhagic stroke (13q34, 17q23.3 OMIM 614519) [5]. Several studies favor a genetic role in the etiopathogenesis of ischemic stroke, including studies of twins [6] and familial aggregation [7].

The role of hypertension in the pathogenesis of stroke has been previously emphasized by findings in meta-analyses and systematic reviews [8,9]. The mechanism of hypertension is complex and it does not only affect the atherosclerosis processes but is associated with a fibrinoid necrosis of small penetrating arteries of the brain [5].

During the thirty years of developing the homozygous recessive characteristics (HRC) test, the authors of the Belgrade population-genetic school selected 15 to 20 as the most reliable characteristics according to the type of inheritance and variability [10,11,12,13,14,15,16,17,18,19]. By establishing the extremely expressed recessive phenotypes, they actually gained insight into the status of these gene loci and the general homozygosity of individuals and groups. These authors studied the distribution and frequency of a series of highly expressed recessive morphophysiological traits in order to estimate individual and group differences (i.e., comparison between ill and healthy individuals, pupils from special and regular schools and carriers of different blood types). Furthermore, previous population genetics studies showed that the number of tested traits was adequate for comparisons of different subpopulation groups [10,11,12,13,14,15,16,17,18,19].

Thus far, there have only been a few population genetic studies focusing on stroke in Serbia. Thus, we created the hypothesis that increased genetic homozygosity along with decreased variability in individuals with manifested ischemic stroke might be populational genetic parameters that can be used to predict ischemic strokes. The aim of our study was therefore to evaluate and compare the morphogenetic variability and the degree of recessive homozygosity in the patients with manifested ischemic stroke.

## 2. Material and Methods

### 2.1. Study Group

The study included 120 patients, who presented with manifested ischemic stroke during the time period of 2014–2017. The diagnosis was established by a board-certified neurologist upon admission of the patient in the hospital. Additionally, 194 healthy individuals without manifested ischemic stroke were assessed (controls). Both individuals from controls and the group with manifested ischemic stroke belonged to the same population (Serbian population) with similar social-economic status and age. Demographic and clinical parameters were taken on examination (gender, age, body mass index (BMI), presence of hypertension and diabetes mellitus, dyslipidemia, family history of hypertension, family history of myocardial infarction (MI), prior percutaneous coronary intervention (PCI) and prior strokes, additionally).

The study was conducted according to the principles of good clinical practice and followed the recommendations of the declaration of Helsinki. The study was approved by the Institutional Review Board of Faculty of Medicine, University of Belgrade.

With regards to the presence of hypertension, the tested individuals were grouped into those with hypertension and those without hypertension. The individuals with any other chronic condition were excluded from the study.

Before inclusion in the study, the patients were informed about study protocol and informed consent was obtained.

To assess the presence of the hypertension, we followed the further recommendations: systolic blood pressure (SBP) ≥ 140 mm Hg or a diastolic blood pressure (DBP) ≥ 90 mm Hg [20].

### 2.2. Tested Determinants

Homozygously recessive characteristics (HRC) test [10,11,12] was conducted for the estimation of the degree of recessive homozygosity in tested subjects. This test was developed for assessing the proportion of clearly expressed homozygously recessive characteristics, which are considered as qualitative traits, in every individual as the markers of chromosomal homozygosities [12,13,14,15,16,17,18]. The tested HRCs are the markers of genes that are located on different chromosomes [19]. Our study included the estimation of the presence of 19 HRCs in the tested individuals where only the characteristics with extreme appearances were marked as the present trait. In the region of the human head, we tested 13 HRCs: attached ear lobe (OMIM number 128900), continuous frontal hair line (OMIM number 194000), blue eyes (gene location 15q12, 15q13, OMIM number 227220; 5p13 OMIM number 227240; 14q32.1, OMIM number 210750; 9q23 OMIM number 612271), straight hair (1q21.3, OMIM number 139450), soft hair and blond hair (gene location 15q12, 15q13, OMIM number 227220; 14q32.1, OMIM number 210750; 12q21.3 OMIM number 611664; 11q13.3, OMIM number 612267), double hair whorl, opposite hair whorl orientation (OMIM number 139400), an inability to roll, fold and curve the tongue (OMIM number 189300), ear without Darwinian notch, ability to produce a guttural “r” and color blindness (gene location Xq28, OMIM number 303800). In human arms, we tested 6 HRCs: proximal thumb hyperextensibility, index finger longer than the ring finger (OMIM number 136100), left-handedness (gene location 2p12-q22, OMIM number 139900), right thumb over left thumb (hand clasping) (OMIM number 139800), top joint of the thumb >45° and three tendons in the wrist (OMIM) [5].

## 3. Statistical Analysis

The obtained results were presented as whole numbers with percentages and as continuous variables as mean value ± standard deviation (MV ± SD). For comparisons of the frequencies of HRCs between the controls and patients with manifested ischemic stroke, we used the Chi-squared test (χ^2^). Mann-Whitney *U* Test and Students *t*-test to assess statistical difference between the tested groups of patients. To quantify the strength of the association of the significant predictors and the presence of ischemic stroke, we calculated the odds ratio (OR) with 95% confidence interval (CI). To compare the variability between the studied groups of individuals, we used the variation coefficient (V). The effect size (Cohen’s d) was used to evaluate the correlation between the tested variables. The statistical significance was set at *p* < 0.05.

## 4. Results

From 120 patients that were diagnosed with manifested ischemic stroke, 64 (53.33%) did not have hypertension and 56 (46.67%) have hypertension.

There were 194 controls, of which 108 (55.67%) have no hypertension and 86 (44.33%) have hypertension. The demographic and clinical parameters are presented in Table 1.

In Table 2, we presented the distribution of HRC frequencies for the controls and patients with manifested ischemic stroke. There were 4 HRCs that significantly differed, of which 3 (blue eyes, inability to transversally tongue roll and right thumb over left thumb) occurred significantly more frequently in patients with manifested ischemic stroke, while 1 (continuous hair line) occurred significantly more frequently in controls. There was a significant difference in the individual variations of 19 HRCs between the controls and patients with manifested ischemic stroke (∑χ^2^ = 60.162; degree of freedom (df) = 18, *p* < 0.01) (Table 2).

The significant predictors of tested HRCs in the studied group of ischemic stroke patients were: soft hair despite the fact that it was not significantly more frequent (OR = 1.72), blue eyes (OR = 3.14), inability to transversally tongue the roll (OR = 2.02) and right thumb over left thumb (OR = 1.86). For controls, this was continuous hair line (OR = 0.44). The most significant predictor for the group of patients with ischemic stroke was blue eyes (Table 2).

**Figure 1:**
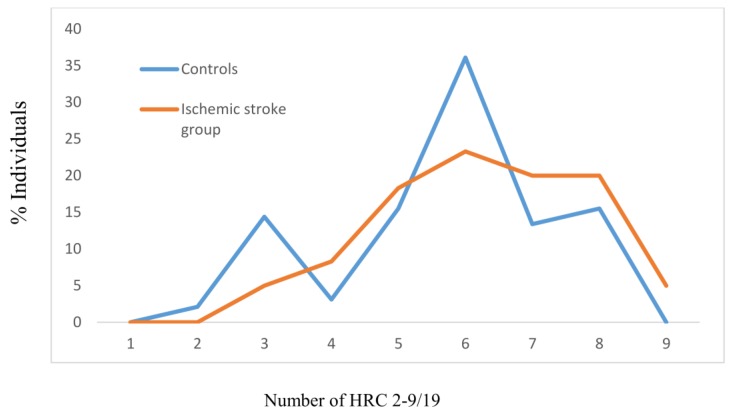
Frequencies of homozygous recessive characteristics (HRC) in controls and manifested ischemic stroke patients. MV- mean value; SD- standard deviation; *z*- Mann Whitney *U* test; V- variability. Controls: *N* = 194, MV ± SD = 5.71 ± 1.61. Ischemic stroke group: *N* = 120, MV ± SD = 6.25 ± 1.54 (*z* = −2.496, *p* = 0.012; Cohen’s *d* = 34.28%). V_Controls_ = 28.20%, V_Ischemic stroke group_ = 24.64%.

The mean values of the tested HRCs significantly differed between the controls and group with manifested ischemic stroke (MV ± SD_Controls_ − 5.71 ± 1.61, MV ± SD_Ischemic stroke group_ − 6.25 ± 1.54, *z* = −2.496, *p* = 0.012), with the effect size between groups being 34.28% (Figure 1). For the group of tested controls, the most frequent average number of HRC was 6 (36.1%), while for those with manifested ischemic stroke, it was also 6 (23.3%) (Figure 1).

**Figure 2:**
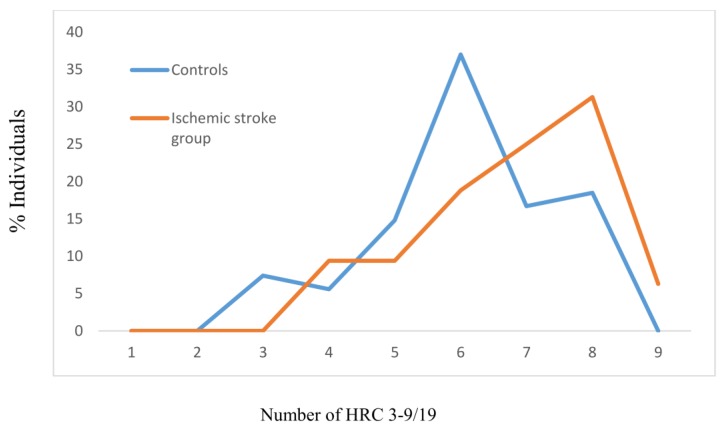
Frequencies of homozygous recessive characteristics (HRC) in controls and manifested ischemic stroke patients without hypertension. MV- mean value; SD- standard deviation; *z*- Mann Whitney *U* test; V- variability. Controls: N = 108, MV ± SD = 6.02 ± 1.40. Ischemic stroke group: *N* = 64, MV ± SD = 6.78 ± 1.40 (*z* = −3.160, *p* = 0.002; Cohen’s *d* = 54.29%). V_Controls_ = 23.26%, V_Ischemic stroke group_ = 20.65%.

For the group of individuals without hypertension, the mean values of HRCs significantly differed between controls and those with manifested ischemic stroke (MV ± SD_Controls_ − 6.02 ± 1.40, MV ± SD_Ischemic stroke group_ − 6.78 ± 1.40, *z* = −3.160, *p* = 0.002), with the effect size between the groups being 54.29% (Figure 2). The most frequent average number of HRCs for the controls without hypertension was 6 (37%) and for those without hypertension and manifested ischemic stroke, this was 8 (31.3%) (Figure 2).

**Figure 3:**
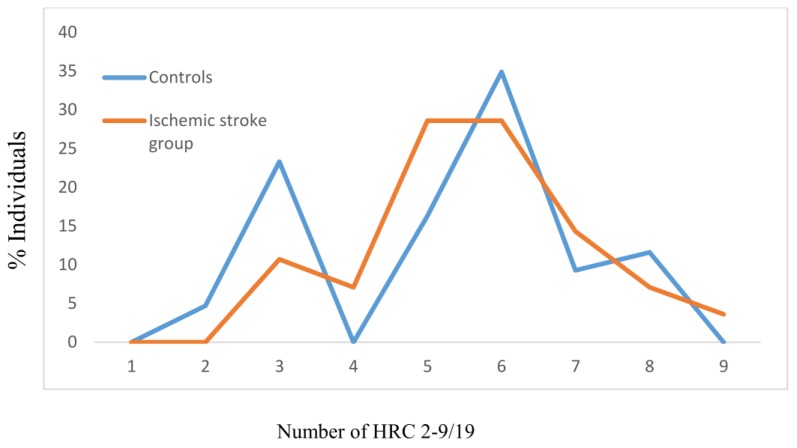
Frequencies of homozygous recessive characteristics (HRC) in controls and manifested ischemic stroke patients with hypertension. MV- mean value; SD- standard deviation; *z*- Mann Whitney *U* test; V- variability. Controls: *N* = 86, MV ± SD = 5.28 ± 1.75. Ischemic stroke group: *N* = 56, MV ± SD = 5.64 ± 1.48 (*z* = −0.783, *p* = 0.435; Cohen’s *d* = 22.21%). V_Controls_ = 33.14%, V_Ischemic stroke group_ = 26.24%.

For the tested individuals with hypertension, the mean values of HRCs did not significantly differ between the controls and those with manifested ischemic stroke (MV ± SD_Controls_ − 5.28 ± 1.75, MV ± SD_Ischemic stroke group_ − 5.64 ± 1.48, *z* = −0.783, *p* = 0.435), with the effect size between groups being 22.21% (Figure 3). The most frequent average number of HRCs in the controls with hypertension was 6 (around 35%). For those with manifested ischemic stroke and with hypertension, this number was between 5–6 (slightly below 30%) (Figure 3).

We found a significant difference in the frequencies of HRCs between those with and without hypertension for controls (*p* < 0.003) and for those with manifested ischemic stroke (*p* < 0.001) (Table 3). The effect size between the groups for the controls was 46.70%, while this was higher for those with manifested ischemic stroke (79.14%) (Table 3).

In Table 4, we presented an association between the frequencies of HRCs regarding the presence of ischemic stroke and hypertension. For controls, 3 HRCs (OR = 3.79) were significant predictors for hypertension. For the ischemic stroke group, 5 HRCs (OR = 3.87) and 8 HRCs (OR = 0.17) were significant predictors for the development of hypertension. For those without hypertension, 6 HRCs (OR = 0.39) and 9 HRCs (OR = 2.74) were significant predictors for ischemic stroke.

## 5. Discussion

Despite the fact that stroke is most frequently considered as a multifactorial disorder where classical patterns of inheritance are hard to demonstrate, there are single gene defects that have been described, which have been predominantly associated with stroke [21]. In the meta-analysis by Hamzi et al., it was stressed that there was an association of some genes with ischemic stroke [22]. Furthermore, another meta-analysis conducted by Casas et al. pointed out that common variants in several genes were associated with an increased risk of stroke, with each having a modest effect [23]. Previously, it was noticed that genes are not only responsible for increased susceptibility to having a stroke but might influence an individual’s response to pharmacological treatment and the ultimate clinical outcome [24]. Moreover, in a multi-ancestry genome-wide study by Malik et al. [25], 32 gene loci were found to be significantly associated with the occurrence of strokes, of which 22 were new discoveries (the location of the gene is on the following chromosomes: 1–7, 9, 10, 12, 13, 15, 17 and 19). This study suggests a wide polygenic determination involved in the expression of the stroke. Therefore, this stresses how heterogeneous and complex the etiology of stroke is. Furthermore, in the era of precision medicine, it was noticed that the polygenic risk core approach is superior to the weighted multi-loci genetic risk score in the assessment of ischemic stroke genetic risks [26].

We have shown that there is a significant difference in the individual variations of tested HRCs between controls and patients with manifested ischemic stroke. Four HRCs were significant predictors in the group with ischemic stroke patients. We noticed that the presence of blue eyes had the highest strength of association with more than 3 times of possibility to be expressed. The presence of a continuous hair line was a significant predictor of stroke in controls, which could have protective effects to the certain degree. These findings suggest that intrinsic changes might exist between these two samples of tested individuals on the population genetic level, assuming that the preferential phenotypes could have certain roles in the development of ischemic stroke. The genes controlling the tested HRCs in this study might have an influence on an individual’s predisposition to the development of ischemic stroke to a certain degree [19].

Furthermore, the findings of this study demonstrated a significantly increased degree of recessive homozygosity in the group of patients with manifested ischemic stroke compared to controls. Despite the fact that the most frequent number of HRCs was 6 in both groups of tested subjects, controls more frequently had this number compared to stroke patients. However, higher numbers of HRCs (indicating the presence of a higher degree of recessive homozygosity) was clearly present in patients with manifested ischemic stroke. Increased recessive homozygosity in the tested group of patients with ischemic stroke might bring the organism into a specific state of genetic-physiological homeostasis, which enables easier expression of this condition [12,19]. Moreover, the increased recessive homozygosity could increase the genetic load, which might influence the processes that lead to a decrease in body immunity, predisposing this organism to developing ischemic stroke [12,27]. Finally, a higher degree of recessive homozygosity in stroke patients might be the result of the pleiotropic effects of genes that are responsible for an individual’s susceptibility to ischemic stroke [12,15].

For the group of tested subjects that did not have hypertension in our study, the patients with manifested ischemic stroke had a significantly higher degree of recessive homozygosity along with decreased variability. Despite the fact that we found no significant differences in the degree of recessive homozygosity between controls and stroke patients with diagnosed hypertension, there is increased recessive homozygosity along with decreased variability in patients with manifested ischemic stroke. These findings might justify the premise that genes determining the evaluated HRCs along with the environmental factors could potentially influence the development and easier expression of manifested ischemic stroke to a certain degree [16]. Our findings are in line with previous reports, which stated that hypertension has a multifactorial origin with both genetic and environmental factors contributing to different degrees [22,23,28]. Therefore, the increased degree of genetic homozygosity in the studied sample of stroke patients with hypertension compared to controls with hypertension implies that preferential phenotypes might exist, which could increase the susceptibility to the easier development of ischemic stroke. Furthermore, the presence of different variations in HRCs between controls and patients with manifested ischemic stroke with and without hypertension might modify the sensitivity of extreme genotype exposures to the risk of being influenced by the processes, which could lead to the onset of ischemic stroke [12].

There was a significant difference in the degree of recessive homozygosity between groups with and without hypertension separately for controls and ischemic stroke patients, with a greater effect size for the group of patients with manifested ischemic stroke. This could provide support for the assumption that there might be a correlation between different combinations of polygenes, which could influence the regulatory processes of resistance to ischemic stroke in stroke patients with hypertension to a certain degree. Furthermore, regarding the presence of hypertension, the different proportion of numbers of HRCs in ischemic stroke group compared to controls was found to be a significant predictor.

The increase in genetic homozygosity is probably correlated with the increase in genetic loads, which may enable easier expression of such condition. Therefore, manifested ischemic stroke patients without hypertension show a higher degree of recessive homozygosity. For the group of patients with hypertension, the association between manifested ischemic stroke and a lower degree of recessive homozygosity also requires the influence of the risk factor.

Furthermore, it should be stressed that the relatively large individual variation in the studied HRCs between controls and group with ischemic stroke, which covers almost all parts of the human body, also provides information about how large this variation in genetic homeostasis can be in human individuals, with a higher chance of individuals with the extreme genotypes having an increased risk of suffering from specific metabolic and developmental malformations. Consequently, the future application of HRC testing can be valuable for predicting these extremely deviant genotypes, which can cause people to be more susceptible to different diseases and conditions.

There are several limitations to this study. The tested HRCs that were evaluated in this study should be improved further as the origin of genetic determination and type of inheritance needs to be better evaluated. Moreover, the pleiotropic effects of the tested HRCs in studied individuals should be also be studied further. Additionally, the pleiotropic causes of risk factors along with tested HRCs should considered in future studies, particularly one using a larger population. This will enable analysis on the population genetic level, which will allow us to obtain a better understanding of the processes that govern the etiopathogenesis of ischemic stroke.

Given the facts above, it can be concluded that the patients with manifested ischemic stroke from our study had an increased degree of recessive homozygosity along with decreased variability compared to controls. The same trend regarding the degree of recessive homozygosity and variability was noticed in the group with hypertension and without hypertension although this occurred to a different degree. The applied methodology potentially might be used in further improved analyses as a sensitive screening tool for early prognosis of susceptibility to ischemic stroke.

## Figures and Tables

**Table 1:** Demographic and clinical parameters of studied individuals.

Parameters		Controls *N* = 194	Ischemic Stroke Group *N* = 120	*p* Values
Gender, *N* (%)	Males	82 (42.27%)	44 (36.67%)	0.325 *
	Females	112 (57.73%)	76 (63.33%)	
Age, years (MV ± SD)		67.49 ± 7.12	71.32 ± 6.92	0.000 **
BMI (MV ± SD)		26.34 ± 8.28	29.14 ± 7.18	0.002 **
Hypertension, N (%)		86 (44.33%)	56 (46.67%)	0.686 *
Diabetes mellitus, N (%)		52 (26.80%)	49 (40.83%)	0.010 *
Non-insulin dependent, N (%)		38 (19.59%)	42 (35.00%)	0.002 *
Insulin dependent, N (%)		14 (7.22%)	7 (5.83%)	0.634 *
Dyslipidemia, N (%)		128 (65.98%)	86 (71.67%)	0.293 *
Family history of hypertension, N (%)		104 (53.61%)	81 (67.50%)	0.015 *
Family history of MI, N (%)		78 (40.21%)	63 (52.50%)	0.033 *
Prior PCI, N (%)		84 (43.30%)	26 (21.67%)	0.000 *
Prior stroke, N (%)		0 (0%)	31 (25.83%)	-

* Chi squared test; ** Students *t* test.

**Table 2:** Frequencies of homozygously recessive characteristics among patients with manifested ischemic stroke and controls.

Homozygously Recessive Characteristics	Controls *N* = 194, *n* (%)	Ischemic Stroke Group *N* = 120, *n* (%)	χ^2^	OR (95% CI)
Blond Hair	52 (26.80)	29 (24.17)	0.258	0.87 (0.51–1.47)
Straight Hair	121 (62.37)	88 (73.33)	1.930	1.64 (0.99–2.69)
Double Hair Whorl	19 (9.79)	12 (10.00)	0.005	1.02 (0.48–2.19)
Opposite Hair Whorl Orientation	40 (20.62)	29 (24.17)	0.611	1.23 (0.71–2.11)
Soft Hair	92 (47.42)	73 (60.83)	3.792	1.72 * (1.08–2.73)
Continuous Hair Line	96 (49.48)	36 (30.00)	7.669 **	0.44 ** (0.27–0.71)
Attached Ear Lobe	31 (15.98)	14 (11.67)	1.162	0.69 (0.35–1.37)
Ear Without Darwinian notch	18 (9.28)	17 (14.17)	2.577	1.61 (0.80–3.27)
Blue Eyes	57 (29.38)	68 (56.67)	25.349 **	3.14 ** (1.95–5.06)
Speaking deficiency -guttural “r”	12 (6.19)	8 (6.67)	0.037	1.08 (0.43–2.73)
Inability to Transversally Tongue Roll	52 (26.80)	51 (42.50)	9.197 **	2.02 ** (1.25–3.27)
Inability to Longitudinally Tongue Roll	73 (37.63)	49 (40.83)	0.272	1.14 (0.72–1.82)
Right Thumb over Left Thumb	97 (50.00)	78 (65.00)	4.500 *	1.86 ** (1.16–2.97)
Top Joint of the Thumb >45°	47 (24.23)	21 (17.50)	1.869	0.66 (0.37–1.18)
Hypermobility of proximal thumb joint	21 (10.82)	14 (11.67)	0.067	1.09 (0.53–2.23)
Proximal thumb extensibility	69 (35.57)	38 (31.67)	0.428	0.84 (0.52–1.36)
Three tendons in the wrist	84 (43.30)	56 (46.67)	0.262	1.15 (0.73–1.81)
Left-handedness	29 (14.95)	16 (13.33)	0.176	0.88 (0.45–1.69)
Index finger longer than the ring finger	86 (44.33)	53 (44.17)	0.001	0.99 (0.63–1.57)
∑χ^2^ = 60.162 **				

* *p* < 0.05; ** *p* < 0.01.

**Table 3:** Statistical evaluation of frequencies of homozygous recessive characteristics between groups with regards to the presence of hypertension.

Groups	With/Without Hypertension		
	Z *	*p*	Cohen’s *d* (%)
Controls	−2.943	<0.003	46.70
Ischemic stroke group	4.037	<0.001	79.14

* Mann Whitney *U* test.

**Table 4:** Association between frequencies of homozygous recessive characteristics regarding the presence of ischemic stroke and hypertension.

No. of HRCs	OR (95% CI)			
	Controls (with/without Hypertension)	Ischemic Stroke Group (with/without Hypertension)	With Hypertension (Ischemic Stroke Group/Controls)	Without Hypertension (Ischemic Stroke Group/Controls)
2	-	-	-	-
3	3.79 ** (1.58–9.10)	-	0.40 (0.15–1.06)	-
4	-	0.74 (0.20–2.78)	-	1.76 (0.54–5.70)
5	1.12 (0.51–2.44)	3.87 ** (1.39–10.73)	2.06 (0.91–4.65)	0.59 (0.22–1.61)
6	0.91 (0.50–1.64)	1.73 (0.74–4.07)	0.75 (0.36–1.55)	0.39 * (0.19–0.82)
7	0.51 (0.21–1.24)	0.50 (0.20–1.28)	1.63 (0.57–4.62)	1.67 (0.78–3.56)
8	0.58 (0.26–1.31)	0.17 ** (0.05–0.53)	0.58 (0.17–1.96)	2.00 (0.98–4.10)
9	-	0.56 (0.10–3.16)	-	2.74 * (1.14–6.62)

* *p* < 0.05; ** *p* < 0.01.
